# The effectiveness of a virtual reality teaching module on advance care planning and advance decision for medical professionals

**DOI:** 10.1186/s12909-023-04990-y

**Published:** 2024-02-05

**Authors:** You-Kang Chang, Yao-Kuang Wu, Tzu-Hung Liu

**Affiliations:** 1 Department of Radiation Oncology, Taipei Tzu Chi Hospital, Buddhist Tzu Chi Medical Foundation, New Taipei City, Taiwan; 2 School of Medicine, Tzu Chi University, Hualien City, Taiwan; 3 Department of Internal Medicine, Taipei Tzu Chi Hospital, Buddhist Tzu Chi Medical Foundation, New Taipei City, Taiwan; 4 Department of Family Medicine, Taipei Tzu Chi Hospital, Buddhist Tzu Chi Medical Foundation, No. 289, Jianguo Road, Xindian District, 231 New Taipei City, Taiwan

**Keywords:** Advance care planning (ACP), Advance decision/directive (AD), Virtual reality (VR), Medical professionals

## Abstract

**Background:**

The concepts of advance care planning (ACP) and advance decisions/directives (ADs) are widely recognized around the world. The Patient Right to Autonomy Act in Taiwan, the first of its kind in Asia, went into effect in 2019. However, a lack of knowledge and confidence regarding ACP and ADs is a barrier for medical professionals in discussing ACP and ADs with their patients. In addition, in Asian countries, physicians tend to make family-centered decisions, which influence how they can implement ADs.

**Methods:**

Virtual reality (VR) is known for its immersive and interactive simulation experience and can upgrade medical education. We developed a VR teaching module to help medical professionals better understand ACP and ADs, with assessment tools integrated into the module. The participants were asked to answer seven knowledge items embedded in the module and fill out the surveys regarding attitudes toward ACP and ADs and confidence in implementing ADs before and after the module. They also reported behaviors related to ADs before and three months after the VR experience.

**Results:**

From July 2020 to June 2022, 30 physicians and 59 nurses joined the study, and 78.7% of them had no prior experience in hospice care. After learning from the VR module, all 89 participants were able to answer all seven items correctly. The results showed a slightly more positive attitude toward ACP and ADs (scores: 32.29 ± 3.80 versus 33.06 ± 3.96, *p* < .05) and more confidence in implementing ADs (scores: 13.96 ± 2.68 versus 16.24 ± 2.67, *p* < .001) after the VR module. Changes in AD-related behaviors (scores: 11.23 ± 4.01 versus 13.87 ± 4.11, *p* < .001) were also noted three months after the VR experience.

**Conclusions:**

This study found that medical professionals may have better knowledge of ACP and ADs, slightly improved attitudes toward ACP and ADs, and greater confidence in implementing ADs after experiencing the VR module. Most importantly, the findings suggested that using a VR format may help motivate medical professionals to perform essential behaviors related to ADs, including introducing ADs to their patients and discussing ADs with their own family.

**Supplementary Information:**

The online version contains supplementary material available at 10.1186/s12909-023-04990-y.

## Background

In recent decades, the concepts of advance care planning (ACP) and advance decisions/directives (ADs) have been widely recognized all over the world. An increasing number of patients expect to receive medical care according to their preferences. Studies have shown that ACP improves the quality of end-of-life care and patient and family satisfaction [1–3]. In Taiwan, the progress in adopting ACP into clinical practice was slow until the Patient Right to Autonomy Act, the first of its kind in Asia, was passed by the Legislative Yuan at the end of 2016 and enacted on January 6, 2019. This Act advocates ADs through the process of ACP counseling. It is aimed at people with any of the following five clinical conditions: terminal diseases, irreversible coma, permanent vegetative state, severe dementia, and other incurable diseases. Several hospitals in different areas of Taiwan have recently reported their experience in inpatient and outpatient ACP services [4–9]. These studies support culturally adapted ACP programs in Taiwan.

Taiwanese people have traditionally taken an evasive and negative attitude toward the issues of death and dying, similar to other Asian countries [10]. For example, Japanese physicians tend to make family-centered decisions, which influence how ADs can be implemented [11]. Among 2,467 cancer patients whose disease was diagnosed as terminal and unresponsive to current curative cancer treatments recruited from 23 teaching hospitals throughout Taiwan, only 7.8% of respondents reported discussing end-of-life care preferences with their physicians in 2014 [12]. Before the Patient Right to Autonomy Act was enacted, a large-scale ACP communication program in Taipei City Hospital showed an increased AD completion rate (82.6%) among inpatients with chronic life-limiting illnesses as a result of the program [4]. The utilization of life-sustaining treatment during the last month of life among older patients decreased following AD completion, which supported the effect of ADs on the utilization of end-of-life treatments [9]. However, a lack of knowledge and confidence regarding ACP and ADs is a barrier for medical professionals in discussing ACP with patients [13–18].

To promote ACP at the national level, patients, medical professionals, and trainees all need to be educated about the importance of ACP [4–11]. We thus need continuing medical education that fits local contexts in Taiwan. In recent years, we have had a 4-hour, lecture-based ACP certification course on legal knowledge and counseling practice for physicians in Taiwan. However, aspects like AD implementation in the clinical settings are not adequately covered by the current 4-hour certification course, and no simulation course was provided. Considering the variety of scenarios in AD implementation, repeatability, and cost-effectiveness in the long run, we chose virtual reality for the simulation course in this study. Virtual reality (VR) is known for its immersive and interactive simulation experience and can upgrade medical education [19, 20]. Empirical studies supported the use of VR to cultivate participants’ empathy in different scenarios [21, 22]. A prior study has shown that engaging people in ACP using a VR experience is feasible [23]. However, there is no research on how we can apply VR in training professionals on ACP and AD implementation. Therefore, we planned to pilot a study using VR for ACP and ADs in Taiwan and evaluate its outcomes for future practice. To exert the potential of VR teaching, we incorporated some core elements from the Social Learning Theory (SLT) proposed by Albert Bandura, including observational learning, modeling, vicarious reinforcement and punishment, self-efficacy, and reciprocal determinism [24]. We hoped to create VR environments that could simulate real-life situations and allow learners to observe and learn from the characters making decisions around ACP and ADs. Learners could watch the characters who demonstrate correct problem-solving and social skills and serve as role models. We also hoped that the consequences of actions and decisions could be simulated in this VR module. When learners saw themselves successfully performing tasks within the VR environment, it could boost their confidence and self-efficacy for similar real-world situations. Finally, the reciprocal interaction between the learner and the VR environment could be leveraged to create personalized learning experiences that adapt to the learner’s decisions and behaviors.

This study had two aims. The first aim was to develop a VR teaching module that could incorporate SLT elements, simulate the situations of AD implementation, and help medical professionals gain a better understanding of ACP and ADs. The second aim was to determine the impact of VR on changing the knowledge, attitudes, confidence, and behaviors towards ACP and ADs.

## Methods

### Development of the VR teaching module

We developed a VR teaching module to help medical professionals better understand ACP and ADs, with assessment tools integrated into the module (See Supplemental Digital Appendix 1). We used the ADDIE instructional design model in the development of our VR teaching module. The ADDIE model stands for analysis, design, development, implementation, and evaluation. In the analysis phase, we conducted the needs assessments, identified the target audience (physicians and nurses), and defined the learning goals and objectives. In the design phase, we outlined the structure and flow of the VR module, defined the assessment methods, and selected the appropriate VR headset and software platform (which was VIVEPAPER® in our case). In the development phase, we organized a group of one instructional designer, two subject matter experts, and two multimedia specialists to create materials and assessments for the VR module. In the implementation phase, we deployed the VR module to the target audience and ensured they could use the VR technology effectively. In the evaluation phase, we collected feedback from the participants and assessed their learning outcomes.

In the VR experience, the participants put on a VR headset, watched a series of 360° videos, and immersed themselves in real-life situations. The participants would observe interactions among the doctor, the nurse, the patient, and his family members from a third-person perspective (aligned with observational learning in SLT) and make clinical decisions from the viewpoint of healthcare providers (aligned with self-efficacy in SLT). This teaching module was set to be 15 to 20 min long. It covered many important issues in ACP and ADs, including AD status, specific clinical conditions defined in the Patient Right to Autonomy Act, health care agents, withdrawal of life-sustaining treatment, and hospice care. There were seven chapters in this VR module, with the first four chapters describing the emergency department setting and the last three describing the ward setting. In each chapter, the participants answered an item in the knowledge domain specific to the chapter, moved on to the next chapter, and saw the characters demonstrating good practice if they answered correctly (aligned with modeling in SLT). If they answered the knowledge item wrong or made a bad clinical decision, the participants received immediate feedback (either in the form of video or text) and were allowed to answer the item again. For example, in the first chapter, the participants decided whether to intubate a patient with impending respiratory failure after reviewing his medical history and AD status. If the participants chose to perform the intubation directly without reviewing the patient’s AD status, they would see the nurse abruptly reminding the physician that the patient had signed the AD during the intubation. This scenario was designed to let participants observe the consequences of unsafe actions and promote the effectiveness of training (aligned with vicarious reinforcement and punishment in SLT). The participants had to answer all seven items in the knowledge domain correctly to finish the whole VR module, and each participant’s VR experience would be slightly different from others based on their choices (aligned with reciprocal determinism in SLT). All these chapters and embedded knowledge items were reviewed and revised by the subject matter experts.

### Evaluation of the VR module effectiveness

The scales used for attitude, confidence, and behavioral domains were modified from a nationwide survey in Japan to fit the contexts in Taiwan [11]. All the participants were invited to fill out the surveys regarding attitudes toward ACP and ADs and confidence in implementing ADs twice. The first assessment took place before the VR experience, and the second assessment occurred immediately after the module. The immediate assessment of attitude and confidence domains was intended to assess how well participants have grasped the materials presented during the module. The scale of attitudes toward ACP and ADs consisted of 9 items (e.g., “My decision in the terminal stage will be respected.”), and the scale of confidence in implementing ADs consisted of 4 items (e.g., “I have confidence in following AD regulations.”). Both scales were 5-point Likert scales. The participants answered the seven items in the knowledge domain related to ACP and ADs again on the follow-up survey three months after the VR experience. They also reported behaviors related to ADs twice. The first assessment took place before the VR experience, and the second assessment occurred three months afterward. The follow-up assessment of knowledge and behavioral domains was intended to assess whether the participants retained their knowledge about ACP and ADs and changed their behaviors in the daily practice after a period of time. The scale of behaviors related to ADs consisted of 5 items (e.g., “I would actively check patients’ AD status.”). The first four items were on a 5-point Likert scale, and the fifth item asked the participants whether they had signed their own AD. Considering the potential burden on participants and their willingness to respond, we did not include the attitude and confidence domains in the follow-up survey. The learner reaction data was also collected based on 4 items and one free-text question immediately after the module. All the above-mentioned survey items were reviewed by five local ACP and AD experts.

### Participants

We launched the VR education module for ADs in mid-2020 in a teaching hospital in northern Taiwan. We adopted voluntary sampling for this study. The inclusion criterion was that the participants should be practicing physicians or nurses in our hospital. The exclusion criterion was that the participants refused to give informed consent. From July 2020 to June 2022, 89 medical professionals joined our study. All of them completed the pretest survey, VR module, and posttest survey immediately after the VR module. Seventy-five of them (84.3%) completed the follow-up survey three months after the VR module. The Institutional Review Board of Taipei Tzu Chi Hospital (No. 09-X-027) granted ethical approval with oral informed consent. We obtained oral informed consent from all the participants.

### Statistical analysis

The demographic characteristics of the participants were described using descriptive statistics. Both the scale of attitudes toward ACP and ADs and the scale of confidence in implementing ADs were tested using Cronbach’s alpha for internal consistency. For all the scale items reviewed by experts, we used the Content Validity Index (CVI) to ensure that the items are relevant for assessing the construct of interest. Both the Item-CVI (I-CVI) and the Scale-CVI (S-CVI), equal to or higher than 0.80, were deemed acceptable. The pretest, posttest, and follow-up survey results were analyzed using Student’s t tests and a chi-square test. The level of significance (alpha) was set as 0.05. Stata/IC version 15.1 (StataCorp. 2017. Stata Statistical Software: Release 15. College Station, TX: StataCorp LLC.) was used for statistical analysis.

## Results

From July 2020 to June 2022, 30 physicians and 59 nurses joined the study, and 70 of them (78.7%) had no prior experience in hospice care. The demographic characteristics of the participants are shown in Table 1. The mean age of physician participants was 36.9 years. Most of them were male (70.0%). Their mean years of experience was 9.5 years. The mean age of nurse participants was 32.7 years. All of them were female. Their mean years of experience was 10.2 years. On their first attempt at the seven knowledge items embedded in the VR module, the participants, on average, answered 4.9 items correctly. After learning from the VR module, all 89 participants were able to answer all seven items correctly.

**Table 1:** Demographic data of the participants (n = 89)

Category	Variable	Mean (SD)	N (%)
Physician (n = 30)	Age (yr)	36.9 (9.6)	
	Sex		M: 21 (70.0%), F: 9 (30.0%)
	Years of experience	9.5 (9.7)	
	Hospice experience		Y: 8 (26.7%), N: 22 (73.3%)
	Clinical specialty	Internal medicine: 5 (16.7%) Surgery: 4 (13.3%) Emergency medicine: 3 (10.0%) Family medicine: 2 (6.7%) Psychiatry: 1 (3.3%) Physical medicine and rehabilitation: 1 (3.3%) Ophthalmology: 1 (3.3%) Otolaryngology: 1 (3.3%) Anesthesiology: 1 (3.3%) General medicine (not yet specialized): 11 (36.7%)	
Nurse (n = 59)	Age (yr)	32.7 (11.1)	
	Sex		M: 0, F: 59 (100%)
	Years of experience	10.2 (9.4)	
	Hospice experience		Y: 11 (18.6%), N: 48 (81.4%)
	Nursing specialty	Internal medicine: 30 (50.8%) Surgery: 19 (32.2%) Outpatient service: 6 (10.2%) Pediatrics: 2 (3.4%) Hospice: 1 (1.7%) Hematology: 1 (1.7%)	

### Attitudes toward ACP and ADs

The first six items of the scale of attitudes toward ACP and ADs indicated positive attitudes toward ACP and ADs, whereas the last three items indicated negative attitudes toward ACP and ADs. The Cronbach’s alpha of the scale of attitudes toward ACP and ADs (with the scores of Items 7 to 9 reversed) was up to 0.88 and indicated that this scale had very good internal consistency. The I-CVIs for these items were all 1.00 except for Item 9 (0.80), and the S-CVI was 0.98. In Table 2, we present the item and scale scores of attitudes toward ACP and ADs. Immediately after the VR module, the participants showed some increase in scores addressing positive attitudes and a decrease in scores addressing negative attitudes (but did not reach statistical significance) except for item 8 (scores: 1.89 ± 0.93 versus 1.58 ± 0.91, *p* < .01). Fewer participants agreed that no more medical services would be provided if they signed ADs. Overall, a slightly more positive attitude toward ADs was noted after the participants experienced the VR module (scores: 32.29 ± 3.80 versus 33.06 ± 3.96, *p* < .05). The subgroup analysis was conducted, and we found that the total score differences in the attitude domain were not as significant among nurses compared to physicians, primarily due to the lack of statistical significance in nurses’ score differences in item 8.

**Table 2:** Item and scale scores of attitudes toward advance care planning and advance decisions (n = 89)

Item	Pretest score Mean (SD)	Posttest score Mean (SD)	*p* value
1. My decision in the terminal stage will be respected.	3.79 (0.55)	3.84 (0.52)	0.167
2. Conflicts between family will be reduced.	3.76 (0.62)	3.75 (0.61)	0.798
3. Futile medical care can be reduced.	3.78 (0.60)	3.84 (0.54)	0.159
4. Unnecessary use of medical resources can be prevented.	3.82 (0.59)	3.84 (0.52)	0.567
5. I will suffer less in the terminal stage.	3.81 (0.58)	3.87 (0.50)	0.167
6. My end-of-life quality will be improved.	3.80 (0.57)	3.88 (0.50)	0.090
7. I feel unsafe about future medical care.	1.97 (0.88)	1.93 (1.13)	0.778
8. No more medical services will be provided.	1.89 (0.93)	1.58 (0.91)	0.003**
9. I can do nothing but wait to die.	1.61 (0.86)	1.45 (0.80)	0.123
Scale	32.29 (3.80)	33.06 (3.96)	0.025*

*Note.* The scale score is the sum of 9 item scores, with the scores of Items 7 to 9 reversed

* *p* < .05, ** *p* < .01

### Confidence in implementing ADs

The Cronbach’s alpha of the scale of confidence in implementing ADs was up to 0.81 and indicated that this scale had good internal consistency. The I-CVIs for all items of the scale of confidence in implementing ADs were 1.00, and the S-CVI was 1.00. Table 3 shows item and scale scores of confidence in implementing ADs. Immediately after the VR module, the participants showed a significant increase in scores on all four items. The participants were more confident in following AD regulations and discussing ADs with their patients. In addition, they were more familiar with hospice care and the Patient Right to Autonomy Act. Overall, more confidence in implementing ADs was noted after the participants experienced the VR module (scores: 13.96 ± 2.68 versus 16.24 ± 2.67, *p* < .001). The subgroup analysis was conducted, and we found that the total score differences in the confidence domain were statistically significant for both physicians and nurses.

**Table 3:** Item and scale scores of confidence in implementing advance decisions (ADs) (n = 89)

Item	Pretest score Mean (SD)	Posttest score Mean (SD)	*p* value
1. Following AD regulations	3.90 (0.81)	4.22 (0.70)	< 0.001*
2. Familiarity with hospice care	3.48 (0.71)	4.10 (0.67)	< 0.001*
3. Confidence in discussing ADs with patients	3.36 (0.86)	3.96 (0.80)	< 0.001*
4. Familiarity with the Patient Right to Autonomy Act	3.21 (0.90)	3.96 (0.74)	< 0.001*
Scale	13.96 (2.68)	16.24 (2.67)	< 0.001*

*Note.* The scale score is the sum of 4 item scores

* *p* < .001

### Knowledge related to ACP and ADs

On the first attempt of the seven knowledge items embedded in the VR module, the participants, on average, answered 4.9 items correctly. After learning from the VR module, all 89 participants were able to answer all seven items correctly. On average, three months after the VR module, the participants answered 5.4 items correctly on the follow-up survey. Although the participants’ knowledge related to ACP and ADs deteriorated three months later, their knowledge scores were still significantly higher than those of the first attempt (scores: 4.92 ± 1.07 versus 5.39 ± 1.17, *p* < .01). The subgroup analysis was conducted, and we found that the total score differences between the first and the third attempts in the knowledge domain were not as significant among nurses compared to physicians.

### Behaviors related to ADs

Table 4 shows the item and scale scores of behaviors related to ADs. The I-CVIs for these items were all 1.00 except for Item 1 (0.80), and the S-CVI was 0.96. Three months after the VR module, we invited all the participants to fill out the survey, but 14 of them dropped out mainly because they left their nursing units (n = 7) or finished their general medicine training (n = 5). The response rate of the follow-up assessment was 84.3%. The 75 participants showed a significant increase in scores on the first four items. The participants would check their patients’ AD status more actively and introduce ADs and hospice care to appropriate patients or families more frequently. They would also be more willing to discuss ADs with their own family or spouse. Regarding the fifth item, we observed an association between their experience of the VR module and their completion of signing ADs (*X*^*2*^(4): 45.13, *p* < .001). Overall, AD-related behavior changes were noted three months after the participants experienced the VR module (scores: 11.23 ± 4.01 versus 13.87 ± 4.11, *p* < .001). The subgroup analysis was conducted, and we found that the total score differences in the behavioral domain were statistically significant for both physicians and nurses.

**Table 4:** Item and scale scores of behaviors related to advance decisions (ADs) (n = 75)

Item	Pretest score Mean (SD)	Posttest score Mean (SD)	*p* value
1. Actively check patients’ AD status	2.99 (1.22)	3.69 (0.99)	< 0.001*
2. Introduce ADs to appropriate patients or families	2.64 (1.24)	3.29 (1.26)	< 0.001*
3. Introduce hospice care to appropriate patients or families	2.71 (1.24)	3.43 (1.19)	< 0.001*
4. Discuss ADs with my own family or spouse	2.89 (1.11)	3.45 (1.12)	< 0.001*
5. I have signed my AD.	No: 15 No, but plan to do so: 52 Yes: 8	No: 16 No, but plan to do so: 45 Yes: 14	< 0.001*
Scale	11.23 (4.01)	13.87 (4.11)	< 0.001*

*Note.* The scale score is the sum of the first 4 item scores

* *p* < .001

### Learner reaction data

In Table 5, we presented the mean scores of the 4 items on a 5-point Likert scale in the learner reaction data. Three of them were 4.60 or higher, except for the item asking whether they felt comfortable wearing the VR devices. The results of the free-text comments were categorized as follows: (1) interesting and innovative (n = 12); (2) high-quality (n = 12); (3) immersive (n = 3); (4) worth promoting (n = 2). Among our participants, none had issues with familiarity regarding VR technology after a brief introduction. We noted that two participants (2.2%) developed moderate motion sickness during their VR experience. However, they could still finish the module after sitting down for the rest of the module.

**Table 5:** Learner reaction data of this VR module (n = 89)

Item	Mean score
1. VR teaching was helpful for my learning on this clinical topic.	4.64
2. I found VR teaching to be more effective than traditional classroom learning for this subject.	4.60
3. The experience of wearing VR devices made me feel comfortable.	4.34
4. I look forward to using VR learning in other clinical topics.	4.61

## Discussion

In this study, we developed a VR teaching module to help medical professionals gain a better understanding of ACP and ADs and hoped to remove barriers to their implementation. Our results suggested that after the VR module, the participants had improved knowledge of ACP and ADs, a more positive attitude toward ACP and ADs, and more confidence in implementing ADs. Moreover, our study participants showed behavioral changes related to ADs, such as introducing ADs to their patients and discussing ADs with their own family, after experiencing the VR module.

The progress in adopting ACP and ADs into clinical practice has been slow in Taiwan. The Patient Right to Autonomy Act was enacted in 2019. A study carried out by Lin et al. just before the enactment of the Act reported a conceptual model of culturally adapted ACP interventions in the Taiwanese context [5]. End-of-life care communication training for health care practitioners to facilitate open discussions with patients is one of the core components requiring greater emphasis when delivering ACP. According to official statistics released by the Ministry of Health and Welfare, Taiwan, 31,139 individuals had completed ACP and signed ADs from the enactment of the Patient Right to Autonomy Act until December 31, 2021. Compared to the entire population (approximately 23 million) of Taiwan, only 0.1% of them have completed ACP. The low ACP completion rate in Taiwan necessitates the development of teaching modules for medical professionals who need to possess sufficient knowledge to engage patients in ACP.

An umbrella review with a focus on the barriers to ACP implementation showed that the main barriers reported by medical professionals were lack of knowledge and skills to carry out ACP, fear of starting conversations about ACP, and a lack of time for discussions [25]. A recent systematic review explored medical professionals’ knowledge of, attitudes toward, and experiences with ACP in South, East, and Southeast Asia [26]. Most studies enrolled in this review indicated Asian medical professionals’ low engagement and late initiation of ACP, although they acknowledged the importance of ACP. They considered ACP difficult to initiate, partly because of their lack of knowledge and skills in ACP, personal uneasiness to conduct ACP, fear of conflicts with family members and their legal consequences, and the lack of a standard system for ACP. Capacity building for ACP in Asia should focus on culturally adapting ACP models concerning the essential role of the family in Asia and education for medical professionals [26].

Enhancing the effects of continuing education programs for ACP and ADs is a crucial step to changing the attitudes and behaviors of medical professionals. In Taiwan, a 4-hour, lecture-based ACP certification course on legal knowledge and counseling practice has been provided to physicians for years. A systemic review reported that for medical professionals, which included medical, nursing, and social work staff, multimodal programs combining initial didactic teaching and role-play simulation tasks with additional activities were most effective and could produce increased ACP activities in medical records [27]. These education programs have already been implemented for ACP service providers such as nurses, social workers, and psychologists in Taiwan. However, no simulation course of AD implementation was developed. Thus, we sought a technology-enhanced learning approach and used VR in our study.

Studies have emphasized that physicians take the leading role as conductors in the ACP process and that nurses are invaluable facilitators in making the voices of patients and family members heard [28]. Many patients and their family members prefer to talk to their physician, who best knows them, and with whom they already have a therapeutic relationship to address difficult and intimate end-of-life issues [29]. Medical professionals who know the patients well are considered the ideal group of people to initiate ACP [30]. In Taiwan, a multidisciplinary team (comprising a physician, a nurse, and a social worker or psychologist) is required for ACP delivery based on local legislation. Therefore, VR teaching materials should be designed for multiple recipients and should help medical professionals clarify their roles in ACP initiation.

The focus of previous education studies that used videos for ACP and ADs was on ACP participants, while education for medical professionals was still lacking. These studies showed that video decision aids could assist ACP participants and improve some ACP-related outcomes [31]. Patients who used a video decision aid were less likely to indicate a preference for cardiopulmonary resuscitation, and video decision aids resulted in greater knowledge related to ACP. A theoretically based multimedia educational program was also found to be effective in assisting ACP implementation and completing ADs among community-dwelling older adults [32].

VR is an innovative educational tool but is rarely used for ACP and AD education. A prior study in southern Taiwan revealed that an ACP decision aid using VR helped the participants clarify their end-of-life care preferences [23]. After viewing the VR video, the preference for not receiving cardiopulmonary resuscitation, life-sustaining treatment, antibiotics, blood transfusion, and artificial nutrition and hydration increased significantly in the VR intervention group. Uncertainty regarding the medical options for people diagnosed with terminal diseases, those in an irreversible coma or permanent vegetative state, and those with severe dementia or other incurable diseases decreased substantially. VR was employed in our study and recognized by participating physicians and nurses for its help in making end-of-life decisions. As we incorporated the elements of SLT in VR teaching, this module demonstrated effectiveness in many levels of the Kirkpatrick model [33]. The Level 1 Reaction data in our study were generally satisfying. The Level 2 Learning data showed improvement in attitudes, confidence, and knowledge domains, and self-reported changes in daily practice were noted in the Level 3 Behavior data. Our study further supported the use of VR in helping medical professionals acknowledge the importance of ACP and implement ADs in their clinical practice.

VR technology can be a powerful tool for applying SLT in education and training. In our module, the core elements of SLT (observational learning, modeling, vicarious reinforcement and punishment, self-efficacy, and reciprocal determinism) could align well with the design. For example, we enhanced the participants’ self-efficacy by providing them opportunities to practice in safe VR environments. We also allowed participants to experience the outcomes of different choices and decisions based on the concept of vicarious reinforcement and punishment. As a result, both physicians and nurses significantly improved their confidence in implementing AD and changed their AD-related behaviors. However, we observed slight differences in item and scale scores in the attitude domain. In the subgroup analysis, only physicians had significantly improved scores of Item 8 (“No more medical services will be provided.”) in the attitude domain. There was also slight knowledge decay 3 months after the VR experience. These findings suggested that besides using the VR module, regularly reviewing the legal content with healthcare professionals and employing other teaching methods to encourage learner reflection may help improve learners’ knowledge of and attitudes toward the relatively complex Patient Right to Autonomy Act.

This study has some limitations. First, it was a single-site study, which might cause concerns about generalizability. However, the hospital in this study was sponsored by government funding for promoting ACP and was one of the leading hospitals in terms of ACP and AD implementation in Taiwan. The VR module could benefit medical professionals in hospitals where ACP service implementation is still in progress. Second, the VR teaching module was designed to fit the contexts and legal status of Taiwan. Future studies using VR for ACP and AD education should consider laws, regulations, and sociocultural contexts. Thirdly, since we did not include the attitude and confidence domains in the follow-up survey, the mechanism of behavioral changes may need further investigation. Finally, prior research has shown increased ACP discussion and documentation after educational interventions [34]. More studies are needed to explore the evidence for improved clinical outcomes after VR training.

## Conclusions

This study found that medical professionals may have better knowledge of ACP and ADs, slightly improved attitudes toward ACP and ADs, and greater confidence in implementing ADs after experiencing the VR module. Most importantly, the findings suggested that using a VR module may help motivate and empower medical professionals to perform essential behaviors related to ADs, including introducing ADs to their patients and discussing ADs with their own family.

### Electronic supplementary material

Below is the link to the electronic supplementary material.

Supplementary Material 1

## Data Availability

The datasets used and/or analyzed during the current study are available in an anonymized format from the corresponding author upon reasonable request.

## Abbreviations

ACP: Advance care planning
AD: Advance decision/directive
VR: Virtual reality
