# Supplementary material for: The effectiveness of a virtual reality teaching module on advance care planning and advance decision for medical professionals
Source: BMC Med Educ. 2024 Feb 5;24:112. doi: 10.1186/s12909-023-04990-y (PMC10845462; doi:10.1186/s12909-023-04990-y)
Supplement: Supplementary file 1 — Supplementary Material 1 [file 12909_2023_4990_MOESM1_ESM.docx]

Supplemental Digital Appendix 1

**The virtual reality (VR) teaching module on advance care planning and advance decision at Taipei Tzu Chi Hospital**

**Name of the lesson plan:** Implementing advance decisions (ADs) in emergency and hospitalization settings

**Author of the lesson plan:** Dr. Tzu-Hung Liu

**Lesson duration:** 15 to 20 minutes

**Teaching objects:** Physicians and nurses

**Learning goals:**

1. To understand the legislative spirit of the Patient Right to Autonomy Act.

2. To become familiar with specific clinical conditions and medical options in Advance Care Planning (ACP).

3. To properly implement ADs in clinical practice.

**Learning objectives:**

1. To be able to understand the five specific clinical conditions of the Patient Autonomy Act and to determine if the patient meets the conditions.

2. To be able to distinguish the priorities of willing persons, relatives, and healthcare proxies in medical decision-making.

3. To be able to describe the process of AD implementation that includes the assessment and intervention performed by specialist physicians and the palliative care team.

**Evaluation targets:**

1. Once the patient is in a life-threatening situation, immediately contact the family and healthcare proxy.

2. Comprehensively assess the patient's physical condition and various diagnoses to determine whether the patient is terminally ill.

3. In terms of irreversible coma, according to the specific clinical conditions of the Patient Autonomy Act, if it is not due to trauma, the patient needs to be observed for more than three months and is then firmly diagnosed by two neurologists.

4. Under the framework of the Patient Autonomy Act, the following priorities apply (in descending order): the will of the willing person that is expressed at the moment when he/she is aware of it, ADs, the opinion of the healthcare proxy, and the opinions of the family members who are not the healthcare proxies.

5. Two specialist physicians are needed to determine whether a patient meets the criteria for a specific clinical condition.

6. The palliative care team needs to be consulted at least two times before life-sustaining treatments and nutrition are accepted or rejected based on the AD.

7. Palliative care should be administered after termination of life-sustaining treatments and nutrition.

**Pre-class instructions:**

After putting on the VR headset, trainees will be able to watch the interaction between the clinical staff and the patient's family members. During the process, they will consider the medical side of the equation or imagine themselves as a physician. Upon scenario transitions, multiple choice questions will be presented on the screen. The trainees will make judgments based on their understanding of the concepts of the Patient Autonomy Act, and their judgments will affect the progression of the scenario. Furthermore, this module will provide the best demonstration of the medical side under the current legal framework. If trainees find that their choices in answering the questions do not fit into the following scenario, they must reflect on whether there is a cognitive gap.

**Lesson plan** (Assessment embedded teaching):

| **Order** | **Content** | **Remarks** |
| --- | --- | --- |
| Chapter one | Background: An 80-year-old male patient, Mr. Chan, is usually conscious but has become a resident of a nursing home due to limited mobility. The patient has diagnoses of hypertension, hyperlipidemia, old stroke, and prostate cancer (currently controlled by hormone therapy). This morning, the nursing staff called an ambulance and took the patient to the emergency room due to the occurrence of unconsciousness, excessive phlegm, shallow and rapid breathing, and decreased oxygen concentration in the blood with an SpO_2_ of 86%.  Emergency nurse: “Doctor, the patient in the second bed of the emergency room is usually conscious but is now not very alert. His wheezing is getting worse. Do you want to go over and take a look first?”  Emergency physician: “Are his vital signs stable? What is his GCS coma index?”  Emergency nurse: “Blood pressure 124/68 mmHg, pulse 112 beats per minute, and respirations 32 breaths per minute. At the beginning, his SpO_2_ was over 70%. After being given oxygen with an NRM oxygen mask, the SpO_2_ only increased up to 88%, and his body temperature was 37.8 degrees. I just evaluated the GCS, and the index was E2M4V3.”  Emergency physician: “He doesn't sound like he’s in good shape. I am coming over to see the patient.”  (The physician performs auscultation.)  Emergency physician: “Please draw blood for a full septic workup and ABG analysis and collect a urine sample for testing. A chest X-ray should also be performed. There are prominent rales in both lungs. Pneumonia combined with acute respiratory failure should be considered. It is time to prepare for intubation.”  Emergency nurse: “Doctor, everything is ready.” |  |
| Assessment one | Question: What should you do now? (Single-choice question)  Options:  1. First, confirm that wishes for hospice care or ADs are documented in the health insurance card and then contact the family.  2. First aid treatment should be administered immediately. | Option 1 is correct. If the correct answer is given, the lesson will move on to Chapter two. |
| Chapter one (Extended) | (If option 2 for assessment one is chosen, the lesson will jump to the following scene.)  Emergency physician: “Huh? Why can't the tube be inserted?”  Emergency nurse: “Doctor, I just forgot to say that an AD seems to be documented in this patient’s health insurance card. Would you like to take a look?”  Emergency physician: “……” (holds out his hands and rolls his eyes)  (The screen turns black, and the lesson returns to assessment one.) |  |
| Chapter two | Emergency physician: “Hold on, did you find a hospice wish or an AD in the health insurance card just now?”  Emergency nurse: “Yes. The patient has signed an AD before.”  Emergency physician: “Have you talked to the patient’s family?”  Emergency nurse: “His wife has passed away. He only has one daughter. His daughter just told me that she is in the hospital parking lot and will be in the emergency room soon. She said that the patient chooses not to have life-sustaining treatments or artificial nutrition according to his AD. However, she had a different idea on this issue and wanted to discuss it with you. Should intubation be performed or not?”  Emergency physician: “Here's the deal. Let's first support the patient with the NRM oxygen mask for a while. We can wait for his daughter for 1–2 minutes while taking a look at the X-ray and ABG results.”  (The patient's daughter enters.)  Patient's daughter: “Are you the emergency physician? What's wrong with my dad? The people at the nursing home just said he was in very bad shape …”  Emergency physician: “Your father appears to have pneumonia combined with acute respiratory failure. He is now in critical condition and needs to be intubated. Do you know that he had an AD documented on his health insurance card?”  Patient's daughter: “Yes, he suffered from prostate cancer. The doctor advised that he could take medication to keep it under control for a long while, but my dad was afraid that it would worsen later, and he didn't want to drag his family down with him. It was at that time that he took me and his cohabitant to the ACP counseling clinic. He also said that he would designate his cohabitant and me as his proxies. She will be here later. My dad said that if he is not in good condition, there is no need for first aid treatment, but I really couldn't bear to agree with this. At that time, I only witnessed the signature, but I did not agree to be his proxy. Doctor, he is the only family member I have left. I really don't know what to do without him.” (covers her face and cries)  (The patient's daughter calls out to the patient, but there is no response.)  Patient's daughter: “My dad is in a coma. Can you please save him first?” |  |
| Assessment two | Question: What is your judgment now? (Single-choice question)  Options:   1. Given that the patient is terminally ill as defined by the Patient Autonomy Act, no emergency treatment is provided. 2. The patient must first be given emergency treatment as he is not a terminally ill patient as defined by the Patient Autonomy Act. | Option 2 is correct. If the correct answer is given, the lesson will move on to the Chapter three. |
| Chapter two (Extended) | (If option 1 for assessment two is chosen, the lesson will jump to the following scene)  Emergency physician: “Your father has a cancer diagnosis and is now suffering from respiratory failure, which is considered terminal. Thus, we have to do what he wants and let him pass away naturally.”  Patient's daughter: “That's not right, is it, doctor? The doctor who treated my dad for prostate cancer said he was well under control for the cancer and could live for years. If treatments are given now, won't he be able to live? How could he be a terminally ill patient? Are you doing something wrong?” (agitated)  Emergency physician: “……” (helplessly looking at the family member)  (The screen turns black, and this error message continues to be displayed.)  According to the regulations of hospice and palliative care, a "terminally ill patient" is defined as a person suffering from a serious injury or illness that has been diagnosed as incurable by two relevant specialists and will progress to the point where death is inevitable in the near future based on medical evidence. Alternatively, physicians can ask themselves or their healthcare team, "Would you be surprised if the patient died within a year?". If the answer is no, the condition is considered terminal.  (The lesson returns to assessment two.) |  |
| Chapter three | Emergency physician: “Your father may not be terminally ill in terms of his prostate cancer. However, his state of consciousness has deteriorated, and this situation is probably due to an infection. We need to treat his lung infection first, and emergency intubation could help him survive the period of respiratory failure.”  (Proxy Auntie enters)  Proxy Auntie: “Hello, doctor. I am a friend of Mr. Chan. The nurse just told me that Mr. Chan is not in good condition and needs to be intubated ……”  Emergency physician: “Yes, Mr. Chan is suffering from pneumonia combined with respiratory failure. We are now preparing to perform intubation for ventilation. Otherwise, his life will be in danger.”  Proxy Auntie: “I'm telling you that I am his healthcare proxy. He told me countless times that no intubation should be performed when he is in bad condition. Mr. Chan and I went to the ACP clinic and signed the documents. Don't let him suffer, okay?”  Emergency physician: “This... it's better for you and his daughter to reach a consensus on the issue.” |  |
| Assessment three | Question: What is your judgment now? (Single-choice question)  Options:  1. Given that the legal status of the patient’s healthcare proxy is higher than that of his daughter, the proxy’s intention should be considered first. Therefore, first aid is not provided.  2. As the only direct blood relative of the patient, his daughter has a higher legal status than the healthcare proxy. Therefore, first aid should be given according to her wishes.  3. Both the proxy and family members cannot override the wishes of the patient at the moment when he/she is conscious. However, this patient has not yet met the specific clinical conditions of the Patient Autonomy Act. In this case, first aid is required. | Option 3 is correct. If the correct answer is given, the lesson will move on to Chapter four. |
| Chapter three (Extended, A) | (If option 1 for assessment three is chosen, the lesson will jump to the following scene.)  Emergency physician: “I'm sorry. The proxy has a higher legal status than the family members. To follow the proxy’s advice, we cannot provide first aid now and must let Mr. Chan pass away naturally. We will do our best to make him as comfortable as possible during the process.”  Patient's daughter: “That's not quite right, is it, doctor? Didn't you just say that he is not considered terminal? If treatments are given now, won't he be able to live? If you don't give first aid now, are you trying to kill my dad? Are you doing something wrong?” (agitated)  Emergency physician: “……” (Helplessly looking at the family member)  (The screen turns black, and this error message continues to be displayed.)  If the patient is not terminally ill, medical practitioners must perform necessary emergency treatment in accordance with medical practice. The above principle has nothing to do with the fact that “under the framework of the Patient Autonomy Act, the healthcare proxy has a higher legal status on expressing opinions than a family member”  (The lesson returns to assessment three.) |  |
| Chapter three (Extended, B) | (If option 2 for assessment three is chosen, this error message will be displayed.)  Under the framework of the Patient Autonomy Act, the following priorities apply (in descending order): the patient’s willingness at the moment when he/she is aware of it, ADs, the opinion of the healthcare proxy, and the opinions of the family members.  (The lesson returns to assessment three.) |  |
| Chapter four | Emergency physician: “The patient signed an AD when he was conscious. At that time, the doctor would have explained to him that “if it’s not terminal, we’ll still have to do what’s necessary”. I believe he understood and agreed to this. Although the patient is suffering from acute pneumonia, he is not yet terminally ill. Therefore, emergency treatment should be given according to the law.”  (The patient’s daughter and Proxy Auntie look at each other speechlessly.)  Patient's daughter: “Auntie, the doctor mentioned just now that the purpose of intubation is to help Dad survive his critical condition, and Dad may recover after that. If no intubation is performed right now, won't he have a chance?”  Proxy Auntie: “I see. Then, do it if you want to perform intubation. But, doctor, if his condition is too serious and he doesn't wake up, won't he have to be intubated for the rest of his life? How can I live with that?” |  |
| Assessment four | Question: The patient is in a state of persistent severe coma not due to trauma, and the specialist needs to determine if the patient meets the specific clinical conditions of the Patient Autonomy Act. In this case, at minimum how long should the patient be observed before the judgement is made ? (Single-choice question)  Options:   1. One month 2. Three months 3. Six months | Option 2 is correct. If the correct answer is given, the lesson will move on to Chapter five. |
| Chapter four (Extended) | (If option 1 or 3 for assessment four is chosen, this error message will be displayed.)  If consciousness cannot be restored due to brain lesions not caused by trauma, the patient must be observed for more than three months. If consciousness cannot be restored due to trauma, the patient must be observed for more than six months. In both cases, after the observation period, specialists will determine whether the patient meets the specific clinical conditions.  (The lesson returns to assessment four.) |  |
| Chapter five | Emergency physician: “If his consciousness does not improve, we can consider removing the tube after three months of observation based on his AD.”  (Both the patient's daughter and Proxy Auntie remain silent.)  Emergency physician: “Okay, if there's no further questions, would you two please leave the room and take a rest? We're here to perform the intubation first.”  (The curtain is closed. The patient is treated with a ventilator. Unfortunately, he suffered two strokes within a week and then fell into a coma. Three months have passed ……)  Ward nurse: “The patient in this bed Mr. Chan has signed an AD. He fell into a coma after his pulmonary infection was treated. The ventilator can't be removed, and it's been more than three months. His healthcare proxy is an aunt who recently inquired if it is possible to extubate the patient so that he can pass peacefully.”  Ward physician: “That's all right, let me consult the hospital about the procedure for handling this kind of patient. Then, can we schedule a family meeting with the auntie and family members? We'll see what we can do.”  Ward nurse: “All right, I'll tell you when I'm done.” |  |
| Assessment five | Question: How is the diagnosis of persistent severe coma not due to trauma to be ascertained in the specific clinical conditions of the Patient Autonomy Act? (Single-choice question)  Options:   1. The diagnosis can be made by a neurology specialist. 2. The diagnosis needs to be made by two specialists, one of whom is a specialist in neurology. 3. The diagnosis needs to be made by two specialists in neurology. | Option 3 is correct. If the correct answer is given, the lesson will move on to Chapter six. |
| Chapter five (Extended) | (If option 1 or 2 for assessment five is chosen, this error message will be displayed.)  To ascertain that the case meets the clinical criteria for a persistent severe coma, the following conditions should be satisfied: the patient cannot regain consciousness due to brain lesions, and this condition must be diagnosed by two neurologists after the observation period.  (The lesson returns to assessment five.) |  |
| Chapter six | (Ward physician, nurse, patient’s daughter, and Proxy Auntie are sitting in the meeting room.)  Ward physician: “Hello, I am Dr. Chang. Thank you for coming to discuss Mr. Chan's condition with us. We are all very concerned about Mr. Chan's condition. However, more than three months have passed, and there is no improvement in his consciousness state and respiratory function. I would like to discuss the following with you: should we follow his AD and terminate life-sustaining measures?”  Proxy Auntie: “Doctor, I said before that it would be better not to perform intubation in the emergency room, but he has not regained his consciousness even now. It's so painful to see him with the tube inserted every day. Can you please let him be relieved?”  Ward physician: “What about his daughter's idea?”  Patient's daughter: “Are you sure that my dad is unlikely to get better, doctor? If there is a chance of recovery, isn't extubation now going to kill him?”  Ward physician: “We've been monitoring his condition closely, but as you can see, his improvement is very limited, and it's hard to keep the tube in all day. We will be very cautious and invite two neurologists to make a definitive diagnosis, but I can tell you that it will be very difficult for your father to regain consciousness.”  Patient's daughter: “I don't want my dad to have more suffering if the doctor is certain that he will not regain consciousness. I'm just worried that any emergencies may occur after the tube is removed. What should we do if he has shortness of breath? Should we let him fend for himself?” |  |
| Assessment six | Question: What do we need to do before life-sustaining treatment is terminated according to the patient’s AD ? (Single-choice question)  Options:   1. The palliative care team needs to be consulted at least once. 2. The palliative care team needs to be consulted at least two times. 3. The patient needs to be transferred to the hospice ward before life-sustaining treatment is terminated. | Option 2 is correct. If the correct answer is given, the lesson will move on to Chapter seven. |
| Chapter six (Extended) | (If option 1 or 3 for assessment six is chosen, this error message will be displayed.)  To determine if life-sustaining treatment should be rejected or terminated, the palliative care team needs to be consulted at least two times, and a palliative care plan must be drafted. Furthermore, termination of life-sustaining treatment can be performed not only in the hospice ward but also in the original care unit.  (The lesson returns to assessment six.) |  |
| Chapter seven | Ward physician: “It's true that some patients don't pass away immediately after the tube is removed, so we have to make good preparations in order to make the end of his life pain-free. Prior to extubation, we would invite the palliative care team to visit him at least twice and draft a follow-up care plan. Please don't worry.”  Patient's daughter: “I did not quite catch it. Does this mean that my dad has to live in the hospice ward? When my dad was conscious, he said that he preferred to die in his hometown. Can we take him home after the tube is removed?” |  |
| Assessment seven | Question: If the family wants the patient to return home to die, what would you do? (Single-choice question)  Options:   1. Let the patient be discharged from the hospital in critical condition after extubation. 2. Provide home hospice or in-home medical care, allowing the team to assist the family members in caring for the patient to the end of his or her life. | Option 2 is correct. If the correct answer is given, the lesson will move on to Chapter eight. |
| Chapter seven (Extended) | (If option 1 for assessment seven is chosen, the lesson will jump to the following scene.)  (The patient is wheezing, struggling to breathe, and moaning continuously.)  Patient's daughter: “Doctor, my dad’s blood pressure is still stable a few hours after the extubation, but he's wheezing more and moaning all the time. Didn't you say we could take him home and let him pass naturally and comfortably?”  Proxy Auntie: “Yes, doctor. Mr. Chan looks so miserable. How can he go home like this?”  Patient's daughter: “Doctor, I can't bear to see my dad is struggling so hard. Let's put the tube back in, shall we?”  Ward physician: “……” (helplessly looking at the family member)  (The screen turns black, and this error message continues to be displayed.)  After life-sustaining medical treatment is rejected or terminated in accordance with the patient’s AD, palliative care should be included on the medical side in order to pursue dignified death of the patient. Conversely, direct discharge from the hospital in critical condition may lead to poor control of the patient's symptoms before death.  (The lesson returns to assessment seven.) |  |
| Chapter eight | Ward physician: “The hospice team in our hospital provides a home palliative care service, where a doctor and a nurse visit the patient at his/her home and provide medications for symptom control. In addition, some doctors in Taiwan are now starting to conduct in-home medical care services. They can visit patients at home every day and when the patient has an acute medical condition. In this way, even if Mr. Chan returns home, there won't be too much pain for him, and you will feel more at ease when taking care of him.”  Proxy Auntie: “Thank you, doctor! If it can be arranged like this, I will feel relieved, and I will be able to live up to what Mr. Chan has consigned to me.”  The patient's daughter: “Thank you, doctors. You've worked hard!”  The show ends. |  |

**Scoring system:**

Each correct answer will be awarded 1 mark, while incorrect answers will be awarded 0 marks, with the total score being 7 marks. Please note that this assessment is only a formative assessment that can assist trainees in debriefing after the teaching experience and should not be included in the workplace assessment criteria.

**Reference:**

The Hospice Foundation of the Republic of China (Taiwan) (March 22, 2019).【ACP】The guidebook of the Advance Care Planning-Petitioner edition (February 17, 2023):

<https://www.hospice.org.tw/sites/default/files/attfiles/%E9%A0%90%E7%AB%8B%E9%86%AB%E7%99%82%E7%85%A7%E8%AD%B7%E8%AB%AE%E5%95%86%E6%95%99%E6%9D%90-20190322-%E6%B0%91%E7%9C%BE%E7%89%88.pdf>
